# Internal training exposure: development and construct validation of an individualised method using heart rate variability

**DOI:** 10.1007/s00421-025-05841-y

**Published:** 2025-06-11

**Authors:** Samrat Sheoran, Antonis Stavropoulos-Kalinoglou, Josh Darrall-Jones, Dan Weaving

**Affiliations:** 1 Centre for Human Performance, Carnegie School of Sport, Leeds Beckett University, Cavendish G08, Headingley Campus, Leeds, LS6 3QT UK; 2 Applied Sports Science and Exercise Testing Laboratory, The University of Newcastle, Ourimbah, NSW Australia; 3 Department of Physical Activity and Sport, Faculty of Arts and Sciences, Edge Hill University, Ormskirk, UK

**Keywords:** DFA-alpha1, Internal dose, Training impulse, Training load, TRIMP

## Abstract

**Purpose:**

The aim was to develop and validate an individualised internal training exposure method by deriving weighting factors for each heart rate (HR) from detrended fluctuation analysis of heart rate variability (DFA-α1) during a graded exercise test.

**Methods:**

Thirty-seven participants (17 females; 32.72 ± 9.26 years; maximal oxygen uptake, $\dot{V}$O_2max_ = 48.32 ± 7.95 mL kg^−1^ min^−1^) completed a step- and a ramp incremental test to measure blood lactate (BLa), DFA-α1, and cardiorespiratory fitness (CRF) variables, i.e. speed at lactate, ventilatory thresholds (LTs/VTs), and $\dot{V}$O_2max_. Exponential fitting of the fractional elevation of HR (ΔHR) with BLa (individualised training impulse; iTRIMP) or DFA-α1 (αTRIMP) generated individualised coefficients for both methods. The TRIMP weightings were interpolated values of BLa or DFA-α1 derived at each ΔHR through coefficients to represent individualised physiological intensity. Principal component regression evaluated the relationship between combined CRF variables and the TRIMP coefficients or weightings.

**Results:**

Large inter-individual variation was observed at the same physiological thresholds (ΔHR at LT_1_/VT_1_ = 0.51–0.83 and LT_2_/VT_2_ = 0.63–0.96), underscoring the need for TRIMP methods to weight ΔHR and account for different exposure at similar intensity. CRF had a *moderate* relationship with coefficients for iTRIMP and αTRIMP methods (*R*^2^_average_ = 0.52–0.67), but a *moderate* to *strong* relationship with their weightings at a fixed ΔHR (*R*^2^_average_ = 0.67–0.78).

**Conclusion:**

αTRIMP is a valid and practically accessible method for quantifying internal training exposure using ECG-based HR monitors, which individualises physiological intensity through DFA-α1-derived weightings among individuals of varied fitness exercising at same percentages of HR.

**Supplementary Information:**

The online version contains supplementary material available at 10.1007/s00421-025-05841-y.

## Introduction

Understanding the rate, magnitude, and changes over time of exercise exposures and responses are considered crucial in optimising desirable performance and health-related outcomes (Jeffries et al. 2021). Exercise exposures have both external and internal dimensions, in which external exposure captures the frequency, type, duration, and rate/intensity of activities an individual completes in a single session or a period of time (Vanrenterghem et al. 2017). In the field, this is often measured via global positioning systems (e.g. speed, distance), or through the amount of resistance, force, and total work done in resistance training (Scott et al. 2016). The internal exposure reflects the extent of homeostatic disturbances (e.g. physiological, morphological, biochemical, or functional) due to the external exposure experienced (Winter and Fowler 2009; Jeffries et al. 2021). However, a given external exposure can lead to different magnitudes and rates of internal exposure within and between individuals at different times due to individual characteristics (e.g. physiological capacity or psychological states) (Jeffries et al. 2021) (see Supplementary Fig. 1). Typically, subjective (e.g. rating of perceived exertion) (Foster et al. 2001) or objective (e.g. heart rate; HR) (Lambert and Borresen 2010) intensity metrics are combined with time spent at those intensities to capture the overall internal exposure during exercise.

HR-based intensity methods such as percentages of maximal heart rate (HR_max_) or heart rate reserve are commonly used in internal exposure measurement (Davis and Convertino 1975; Jamnick et al. 2020). These methods assume that inter-individual physiological responses are homogenous when exercising at fixed HR percentages. However, given the intrinsic physiological variability that exists between individuals at the same HR (Burnley and Jones 2018; Iannetta et al. 2020), these methods lack appropriateness in capturing individualised intensity and overall internal exposure. To consider this, Banister and Calvert (1980) developed a training impulse method (bTRIMP) with weighted intensity (HR × weighting factor). This exponential weighting factor attempts to represent ‘true intensity’ based on the exponential rise in blood lactate (BLa) concentration with respect to fractional elevation of HR (Banister and Calvert 1980). Whilst sex-specific weighting factors were developed in bTRIMP, these were determined through coefficients which were constant within males and females, thus failing to account for inter-individual variability. Manzi et al. (2009a, b) proposed the individualised TRIMP method (iTRIMP) wherein the weighting factor is determined from individuals’ directly measured BLa and HR relationship during an incremental test. This attempts to individualise the calculated internal exposure at a theoretical standardised intensity (HR) and duration, such that individuals with lower CRF would have higher weightings and therefore greater overall internal exposure. However, to our knowledge, no studies have established this aspect of construct validity for iTRIMP. In addition, this method requires access to lactate-based measurement tools and frequent reassessment, limiting its practical applicability to a wider range of people who partake in exercise. Therefore, the development of more feasible methods is needed that balance validity and practicality.

Technological advancements now allow continuous electrocardiogram (ECG) signals to determine beat-to-beat variation in HR or heart rate variability (HRV) which can serve as a non-invasive indicator of autonomic nervous system (ANS) balance (Borresen and Lambert 2008). The ANS acts as an important regulator of homeostasis during exercise; approaches that measure the ANS-mediated physiological transitions with altering intensity (Sandercock and Brodie 2006; Shiraishi et al. 2018) can possibly represent an individualised measure of weighted training intensity like BLa, yet be practically feasible. Recently, a non-linear dynamics quantification method to examine the correlation properties of HRV within the fractal scale, known as alpha-1 of detrended fluctuation analysis (DFA-α1), has been proposed as a potential measure for real-time assessment of exercise intensity (Rogers and Gronwald 2022). Based on its fractal signals theoretical background, DFA-α1 has primarily been used to demarcate aerobic and anaerobic thresholds based on discrete points of 0.75 and 0.5 in its signal. Recent studies have reported DFA-α1 thresholds having *moderate* to *nearly perfect* agreement (*R* = 0.31–0.96) with first ventilatory/lactate thresholds (VT_1_/LT_1_), but a *high* to *very-high* agreement (*R* = 0.62–0.90) with second ventilatory/lactate thresholds (VT_2_/LT_2_) (Rogers and Gronwald 2022; Schaffarczyk et al. 2023; Fleitas-Paniagua et al. 2024; Sheoran et al. 2024). These mixed findings with unexplained variance in agreement between thresholds and the observed inflated limits of agreement can be attributed to the complexity of underpinning physiological mechanisms (e.g. lactic acidosis, hyperpnoea, and ANS control) which may be causally related to trigger various intensity thresholds, yet have their individual time course. Therefore, it is likely improbable to observe a *perfect* agreement between two measurements at specific points in time.

Given that DFA-α1 exhibits a reverse sigmoidal relationship with increasing exercise intensity unique to every individual, along with the emerging evidence suggesting its association with metabolic thresholds, it is very likely to be representing an underlying intensity-dependent physiological response during exercise. However, a method using the entire time series of DFA-α1 during a graded exercise test as a measure of training exposure, rather than determining specific exercise thresholds at singular points in time, has yet to be explored. Therefore, the primary aim of this study was to develop a practical, non-invasive, and individualised TRIMP method based on weighting factors generated from HRV derived DFA-α1 (referred to here as αTRIMP; see *Methods*). To examine the construct validity of the TRIMP methods, the secondary aim was to investigate the relationship between the weighting factors generated by iTRIMP and αTRIMP with CRF characteristics. We hypothesised that individuals with lower CRF would exhibit higher TRIMP weightings at the same relative HR.

## Methods

### Participants and experimental design

The data reported in the present study was collected as part of study that aimed to investigate the agreement between DFA-α1, ventilatory, and lactate-derived thresholds (Sheoran et al. 2024). Thirty-seven participants (20 males, 17 females; mean ± standard deviation, age: 33 ± 9 years; height: 1.74 ± 0.08 m; body mass: 74.78 ± 17.71 kg; body mass index: 24.49 ± 4.42 kg m^−2^) participated in the study. An exercise pre-participation health screening questionnaire based on American College of Sports Medicine (ACSM) Guidelines for Exercise Testing and Prescription (10th ed.) screened participants for any absolute and relative contraindications or signs and symptoms of diseases (Riebe 2018). All participants cleared screening to perform maximal exercise testing and were physically active (per ACSM criteria of at least 30 min of moderate-intensity physical activity on at least 3 days per week for at least the last 3 months). Participants whose overall RR intervals artifact percentage exceeded 3% (*n* = 4), and whose DFA-α1 versus HR signal attained both thresholds (0.75 and 0.5) multiple times (*n* = 2) within the same continuous incremental test, were excluded for the generation of weighting factors. Therefore, 31 participants were used in the analysis of the present study. The study involved two experimental trials with at least 72 h between them. The first trial involved lactate profiling for calculation of iTRIMP weightings from a step incremental treadmill test. This trial included collecting continuous HR and fingertip-based BLa data at rest during 3-min incremental stages of the treadmill protocol. The second trial involved continuous recording of beat-to-beat RR interval data to generate HRV-based αTRIMP weightings during a continuous incremental ramp treadmill protocol until maximal volitional exhaustion. Lactate-derived CRF measures (speeds at LT_1_ and LT_2_) and ventilator-derived CRF measures ($\dot{V}$O_2max_, speeds at VT_1_, VT_2_ and $\dot{V}$O_2max_) were also determined from step incremental and ramp incremental protocols, respectively. Participants avoided any vigorous physical activity, caffeine, and alcohol consumption 24 h prior to both sessions. Prior to the first trial, participants also provided an informed written consent and anthropometric measurements were taken. The ethical approval of study was granted by Carnegie School of Sport Research Ethics Committee of Leeds Beckett University (Ref: 115967).

### Step incremental treadmill protocol and blood sampling

The step incremental protocol involved 3 min stages on an indoor treadmill (Ergo ELG 2, Woodway, Germany), interspersed with 1 min rest for blood sampling and rating of perceived exertion assessment (RPE; Borg-20 scale). A 20 μL fingertip blood sample was collected, and the BLa concentration was determined using an enzymatic–amperometric based analyser (Biosen C-Line analyzer, EKF diagnostics, Germany). The gradient was kept constant at 1%. Based on the Sport and Exercise Physiology Testing Guidelines by the British Association of Sport and Exercise Sciences, the initial speed was set at ~ 6 km h^−1^ below participants’ recent 10 km performance which enables an ideal number of approximately seven stages to profile both lactate thresholds. A standard starting speed of 6 km h^−1^ was used if participants were uncertain about the pace or were not physically active. Treadmill speed was increased by 1 km h^−1^ each stage (Manzi et al. 2009b; Akubat and Abt 2011) with the test terminating after the stage when both criteria were fulfilled: participants achieving BLa concentration > 4 mmol L^−1^ (Manzi et al. 2009b) and RPE ≥ 18. In instances where one of the criteria was met first, the stage progressions were continued until the second criteria was also achieved. This ensured that both lactate thresholds were attained and a complete BLa-HR profile for all participants could be generated.

Participants wore a chest belt (H10, Polar Electro Oy, Kempele, Finland; sampling rate: 1000 Hz), wirelessly paired to a Polar sports watch (Vantage V, Polar Electro Oy), and the HR data was directly uploaded to the Polar Flow database. The.csv files for every session were exported from Flow for further analysis. The last 30 s time-average HR data during the 3-min stages was used for the determination of fractional elevation in HR (ΔHR) for every stage.

### Blood sampling-based cardiorespiratory fitness (CRF) variables

Lactate thresholds were determined using an online web application (Lactate Thresholds App; https://www.exphyslab.com/lactate), which was supported by the *lactater* R package (Mattioni Maturana 2023). The first lactate threshold (LT_1_) was calculated by the log–log method, as previously described (Beaver et al. 1985). LT_2_ was determined as the speed at which BLa increased 1.5 mmol L^−1^ above participants’ baseline (lowest data point in all stages), as it is observed to be a valid estimate of maximal lactate steady state in a step incremental graded exercise test of 3-min stages (Jamnick et al. 2018). The treadmill speeds corresponding to the LT_1_ and LT_2_ (vLT_1_ and vLT_2_) were used for further analyses as CRF variables.

### Ramp incremental treadmill protocol and DFA-α1 analysis

The ramp incremental protocol was a continuous incremental test performed on an indoor treadmill (Ergo ELG 2, Woodway, Germany). Initial speed was set at a standard 4 km h^−1^, and a constant gradient at 1%, and increased by 0.5 km h^−1^ every 30 s until volitional exhaustion. Participants wore a chest belt (H10, Polar Electro Oy, Kempele, Finland; sampling rate: 1000 Hz) wirelessly paired to a Polar sports watch (Vantage V, Polar Electro Oy), which directly uploaded RR intervals and HR data for the entire protocol onto a Polar Flow database. This was further linked to Kubios HRV Scientific software Version 4.0.3 (Biosignal Analysis and Medical Imaging Group, Department of Physics, University of Kuopio, Kuopio, Finland), where Flexible and Interoperable Data Transfer (FIT) format HRV datafiles were imported for DFA-α1 analysis. The pre-processing settings and analysis methods used for DFA-α1 calculation within Kubios HRV software have been described elsewhere (Schaffarczyk et al. 2023; Sheoran et al. 2024). Post-processing the DFA-α1, .csv files containing DFA-α1 and HR values calculated for 5 s intervals were exported. Supplementary Fig. 2 displays a schematic of all the pre- and post-processing steps for HR and RR intervals data during TRIMPs generation process. The processing of HR data and the exponential modelling of weighting factors with ΔHR were consistent in both the iTRIMP and αTRIMP methods.

### Gas exchange-based cardiorespiratory fitness (CRF) variables

Thirty seconds time-averaged.xml data files of the ramp incremental test from the metabolic cart system (Metalyzer 3B; Cortex Biophysik GmbH, Leipzig, Germany) were exported. The achievement of $\dot{V}$O_2max_ was confirmed when two of the following three criteria were fulfilled: no change in $\dot{V}$O_2_ (< 0.2 L∙min^−1^) despite increasing treadmill speed for at least three 30 s stages, respiratory exchange ratio > 1.1, or reaching 95% age-predicted HR_max_ (Akubat and Abt 2011; Lee and Zhang 2021). The $\dot{V}$O_2max_ was identified as the highest observed value in 30 s time-averaged data during the test. The velocity at $\dot{V}$O_2max_ (v$\dot{V}$O_2max_) was recorded as the minimum treadmill speed that elicited $\dot{V}$O_2max_ over a period of 30 s.

VT_1_ was identified by a combined three determination approach of using modified v-slope method (intersection of two-line regression between $\dot{V}$CO_2_ and $\dot{V}$O_2_ graph), ventilatory equivalencies (E/$\dot{V}$O_2_ nadir or first rise with no concomitant increase in $\dot{V}$E/$\dot{V}$CO_2_ with increasing HR), and end-tidal pressure (P_ET_O_2_ nadir of first increase with increasing HR) (Binder et al. 2008). VT_2_ was identified using respiratory compensation point (inflection point in $\dot{V}$E and $\dot{V}$CO_2_ graph), ventilatory equivalencies ($\dot{V}$E/$\dot{V}$CO_2_ nadir or non-linear rise with increasing HR), and end-tidal pressure (deflection point in P_ET_CO_2_) methods (Binder et al. 2008). Treadmill speeds at VT_1_ and VT_2_ (vVT_1_ and vVT_2_) were determined.

### Calculation of TRIMPs

#### Individualised TRIMP (iTRIMP)

The iTRIMP method utilises the fractional elevation in HR (ΔHR, Eq. 1), training duration accumulated at each ΔHR, and an individualised weighting factor (*y*_*i*_, Eq. 2) which weights ΔHR to its relative exercise intensity.

1 $$\Delta {\text{HR}} = \frac{{{\text{HR}}_{{{\text{exercise}}}} { } - {\text{HR}}_{{{\text{rest}}}} }}{{{\text{HR}}_{\max } - {\text{HR}}_{{{\text{rest}}}} }},$$

2 $$\left[ {{\text{BLa}}} \right]{\text{or }} y_{i} = a_{i} \cdot e^{{\left( {b_{i} \cdot \Delta {\text{HR}}} \right)}} ,$$

*y*_*i*_ is generated by fitting an exponential model to the individual’s BLa response curve during an incremental test with increasing exercise intensity (BLa versus ΔHR) (2), where *a*_*i*_ and *b*_*i*_ are the individuals’ constants and *e* is the base of Napierian logarithm. The intercept coefficient *a*_*i*_ represents resting BLa concentration (i.e. when ΔHR = 0), whereas slope coefficient b_i_ represents the rate of change in BLa concentration with increasing ΔHR.

3 $${\text{iTRIMP}} = \mathop \smallint \limits_{0}^{1} \left[ {{\text{BLa}}} \right] \cdot d\left( {\Delta {\text{HR}}} \right)$$

Overall, the iTRIMP for a given session is calculated as area under the curve or the pseudo-integral of all ΔHR data points (3) (Manzi et al. 2009a).

#### DFA-α1 TRIMP (αTRIMP)

The αTRIMP method uses ΔHR and time spent at each ΔHR as the primary exercise variables, similar to the iTRIMP method. However, the individual weighting factor (*w*_*i*_) is generated by fitting an exponential model to individual’s DFA-α1 response with ΔHR during an incremental test with increasing external intensity. Irrespective of individual fitness characteristics, there is an inverse relationship between DFA-α1 and ΔHR, and a varying nature of the highest or lowest DFA-α1 values individuals attain at rest or maximal exercise intensity, respectively. Therefore, the DFA-α1 trace is first normalised to a scale of 0–1, wherein 0 corresponds to the highest DFA-α1 value at rest (DFA_rest_), while 1 corresponds to the lowest DFA-α1 value at individuals’ maximal intensity during the test (DFA_maximal_). For any given time instant ‘*t*’, and DFA-α1 at t (DFA_t_), the normalised DFA-α1 (or α_norm_) for that instant is given by (4):

4 $${\text{DFA}}_{{{\text{norm}}}} {\text{or }}\alpha_{{{\text{norm}}}} = \frac{{{\text{DFA}}_{{{\text{rest}}}} - {\text{DFA}}_{t} }}{{{\text{DFA}}_{{{\text{rest}}}} - {\text{DFA}}_{{{\text{maximal}}}} }}$$

Following the normalisation of DFA-α1, exponential fitting of α_norm_ (all data points except 0, as ${e}^{x}\ne 0$) versus ΔHR is performed (5), where *p* = 0.06 and q_i_ are individuals’ constants and e is the base of Napierian logarithm. A constant value of *p* was determined as the average of all individual *p*_*i*_ coefficients generated following the exponential fitting (*p*_*i*_ ·*e* ^(*qi* · ΔHR)^) of α_norm_. As the α_norm_ ranges in a bounded scale of 0–1, the intercept (*p*_*i*_), i.e. between-participant DFA-α1 value at rest (ΔHR = 0), was also standardised as a constant *p*.

Similar to iTRIMP, the αTRIMP for a given session is therefore calculated as the pseudo-integral of all ΔHR data points (6).

5 $$\left[ {\alpha_{{{\text{norm}}}} } \right]{\text{or }} w_{i} = p \cdot e^{{\left( {q_{i} \cdot \Delta HR} \right)}} , {\text{where }} p = 0.06,$$

6 $$\alpha {\text{TRIMP}} = \mathop \smallint \limits_{0}^{1} \left[ {\alpha_{{{\text{norm}}}} } \right] \cdot d\left( {\Delta {\text{HR}}} \right)$$

Equations (1) and (2) were used to determine iTRIMP weightings and individual coefficients from step incremental test, while Eqs. (1), (4) and (5) were used to determine αTRIMP-based weightings and coefficients from ramp incremental test.

### Rate of change in iTRIMP and αTRIMP weightings with intensity

The rate of change (slope) of the BLa curve (iTRIMP weightings) with respect to ΔHR was determined by differentiation of Eq. (2),

7 $$\frac{{{\text{d}}y_{i} }}{{{\text{d}}\left( {\Delta {\text{HR}}} \right)}} = b_{i} \cdot \left( {a_{i} \cdot e^{{\left( {b_{i} \cdot \Delta {\text{HR}}} \right)}} } \right)$$

Substituting Eq. (2) in (7),

8 $$\frac{{{\text{d}}y_{i} }}{{{\text{d}}\left( {\Delta {\text{HR}}} \right)}} = {{b}}_{{\varvec{i}}} \cdot y_{i} , {\text{where }} y = \left[ {{\text{BLa}}} \right]$$

Similarly, the slope of α_norm_ curve (αTRIMP weightings) with respect to ΔHR is given by derivative of Eq. (5) or

9 $$\frac{{{\text{d}}w_{i} }}{{{\text{d}}\left( {\Delta {\text{HR}}} \right)}} = q_{i} \cdot \left( {p \cdot e^{{\left( {q_{i} \cdot \Delta {\text{HR}}} \right)}} } \right)\;{\text{or}} = {\varvec{q}}_{{\varvec{i}}} \cdot w_{i} , {\text{where }}w = \left[ {\alpha_{{{\text{norm}}}} } \right].$$

### Statistical analysis

In this study, the independent variables were CRF variables (vLT_1_, vLT_2_, vVT_1_, vVT_2_, $\dot{V}$O_2max_, and v$\dot{V}$O_2max_), and dependent variables were (i) TRIMP weightings intercept coefficients (*a*_*i*_ for iTRIMP and p_i_ for αTRIMP), (ii) slope coefficients (*b*_*i*_ for iTRIMP and q_i_ for αTRIMP), and (iii) overall TRIMP weightings (BLa and α_norm_) at fixed ΔHR of 0.5 and 0.75. To investigate the relationship between CRF and the TRIMP weightings coefficients (*a*_*i*_, *b*_*i*_, *p*_*i*_, and *q*_*i*_), a linear principal component regression (PCR) was performed. The relationship between CRF and TRIMP weightings (BLa and α_norm_) was assessed by a second-order PCR and the model fit was evaluated using tenfold cross-validation.

### Principal component analysis (PCA) of CRF variables

Due to the presence of multicollinearity within CRF variables (vLT_1_, vLT_2_, vVT_1_, vVT_2_, $\dot{V}$O_2max_, and v$\dot{V}$O_2max_; variance inflation factors: 4.91 to 12.26), PCA was performed to reduce the data in the set of uncorrelated principal components (PCs) using *prcomp()* function within the *stats* R package. The suitability of data to perform PCA was confirmed by using Kaiser–Meyer–Olkin (KMO) measure of sampling adequacy and the Bartlett test of sphericity. The KMO value of sampling adequacy was 0.9 for CRF variables, which is over the threshold of KMO ≥ 0.5 suggested to indicate the suitability of data to perform PCA. Bartlett test of sphericity was also significant for the fitness variables (*p* < 0.001). A multifaceted approach which includes the examination of the scree plot, eigenvalues, and the accounted variance by each PC, as recommended by Hair et al. (2009), was implemented to extract the meaningful PCs. The first PC (PC1) captured the most information (88.83% of explained variance) and was extracted for further analysis. PC1 scores for each participant observation (mean centred and standardised vLT_1_, vLT_2_, vVT_1_, vVT_2_, $\dot{V}$O_2max_, and v$\dot{V}$O_2max_ multiplied by their respective PC1 eigenvector) were used as a singular merged CRF measure (CRF_m_) in the following regression models.

### Principal component regression (PCR) between CRF_m_ and TRIMP weightings

PCR models to assess the relationship of intercepts (*a*_*i*_ and *p*_*i*_) and slopes (*b*_*i*_ and *q*_*i*_) of BLa and *α*_norm_ with CRF_m_ were built using the *lm()* function within the *stats* R package. To assess the relationship of iTRIMP and αTRIMP weightings (BLa and α_norm_, respectively) with CRF_m_ at fixed ΔHR of 0.5 and 0.75, four separate second-order PC regression (PCR) models were built. Full model specifications are provided in Supplementary Table 1. The PCR model performance was estimated using K-fold cross-validation using *caret* R package (Kuhn 2008). The *trainControl()* function was set with resampling method as ‘cv’ (cross-validation) and number of folds as ‘10’ for K-fold cross-validation. Finally, the *train()* function was used to estimate the PCR model performance by fitting predictive models on the training sets generated from *trainControl()* function. The average (± SD) coefficient of determination (R^2^_average_) calculated from tenfold cross-validation for each of the PCR models was interpreted similarly like *R*^2^, with values < 0.3 *very weak*, 0.3–0.5 *low*, 0.5–0.7 *moderate,* and > 0.7 *strong* effect size (Moore et al. 2013).

## Results

Table 1 provides the descriptive statistics of all six CRF variables on which PCA was performed. Supplementary Fig. 3 highlights the scree plot with six PCs and their respective eigenvalues. Based on the multifaceted approach to extract meaningful PCs, PC1 (referred to as CRF_m_) captured the most variance and was retained for the regression analysis with TRIMP weightings. The CRF_m_ served as a single merged fitness variable for models, composed of all six CRF variables with their respective loadings.

**Table 1:** Descriptive statistics of all cardiorespiratory fitness (CRF) variables

CRF variable	Mean ± SD [range], *n* = 31
$\dot{V}$O_2max_ (mL kg^−1^ min^−1^)	48.32 ± 7.95 [32.69–68.19]
v$\dot{V}$O_2max_ (km h^−1^)	15.87 ± 2.24 [11.5–21]
vLT_1_ (km h^−1^)	9.83 ± 2.21 [5.5–14.4]
vLT_2_ (km h^−1^)	11.71 ± 2.29 [6.4–16.3]
vVT_1_ (km h^−1^)	10.07 ± 2.11 [6.5–15.2]
vVT_2_ (km h^−1^)	13.28 ± 2.01 [9.2–18.2]

Values are presented as group means ± standard deviation (SD)

$\dot{V}$*O*_*2max*_ maximal oxygen uptake, *v*$\dot{V}$*O*_*2max*_ minimum treadmill velocity to elicit $\dot{V}$O_2max_, *vLT*_*1*_ velocity at the first lactate threshold, *vLT*_*2*_ velocity at the second lactate threshold, *vVT1* velocity at the first ventilatory threshold, *vVT2* velocity at the second ventilatory threshold

**Fig. 1:**
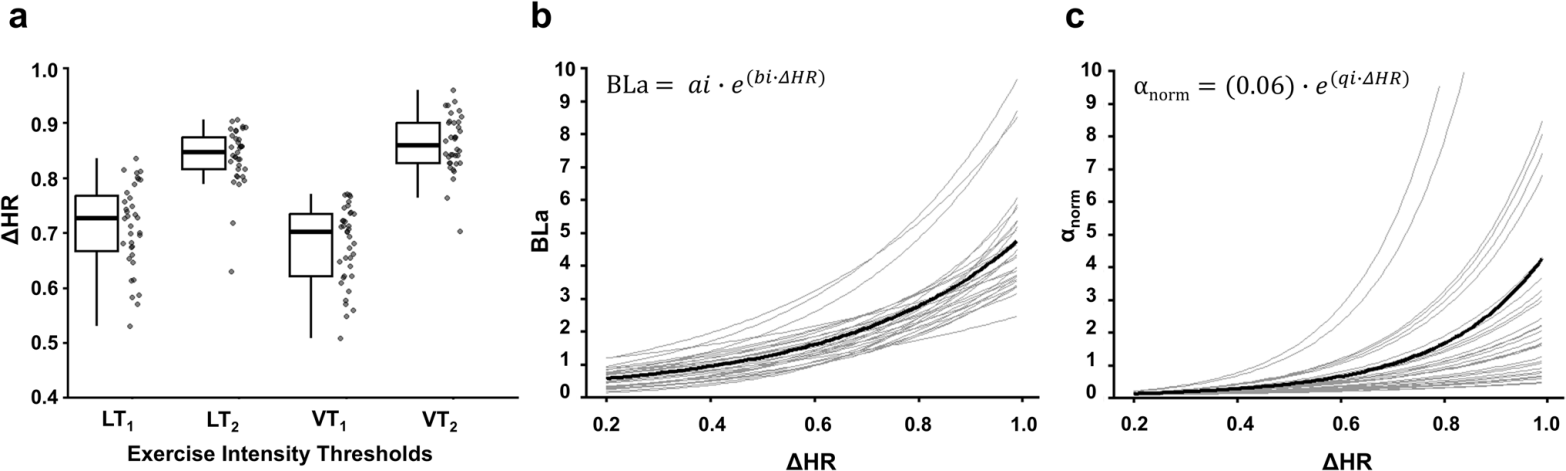
Plots representing **A** between-participant variability in ΔHR at lactate and ventilatory thresholds, **B**, **C** individual (grey lines) and average (black line) BLa and normalised DFA-α1 responses with ΔHR. Abbreviations: ΔHR, fractional elevation in HR; *LT1,* first lactate threshold; *LT2,* second lactate threshold; *VT1,* first ventilatory threshold; *VT2,* second ventilatory threshold; *BLa,* blood lactate; *α*_*norm*_, normalised DFA-α1

Figure 1A displays the inter-individual variability that exists in internal intensity (ΔHR) at defined intensity thresholds, i.e. LTs and VTs (ΔHR; mean ± SD [range], LT_1_ = 0.71 ± 0.08 [0.53–0.83], LT_2_ = 0.84 ± 0.05 [0.63–0.91], VT_1_ = 0.68 ± 0.07 [0.51–0.77], VT_2_ = 0.86 ± 0.05 [0.70–0.96]). Figures 1B, C indicates the individual BLa and α_norm_ profiles generated for the determination of weighting factors at each ΔHR for every participant. The individual profiles express the variability of internal responses at same ΔHR and account for these differences as weightings factors within TRIMPs.

Supplementary Table 1 describes the CRF_m_ models and their performance for individual TRIMP coefficients and the generated weightings. CRF_m_ significantly predicted the individual coefficients *a*_*i*_, *b*_*i*_, *p*_*i*_, *q*_*i*_ (*β*_PC1_ = – 0.28 to – 0.05, 0.01 to 0.12), where *a*_*i*_ and *p*_*i*_ coefficients reflect the resting levels of BLa and *α*_norm_, respectively (i.e. at ΔHR = 0), while b_i_ and q_i_ coefficients indicate the rate of change in BLa/*α*_norm_ with increasing ΔHR. BLa and α_norm_ based weighting factors at ΔHR of 0.5 and 0.75 were also significantly predicted by CRF_m_ (*β*_PC1_ = – 0.43 to – 0.09, *β*_PC1_^2^ = 0.01 to 0.10) with similar model fit parameters.

**Fig. 2:**
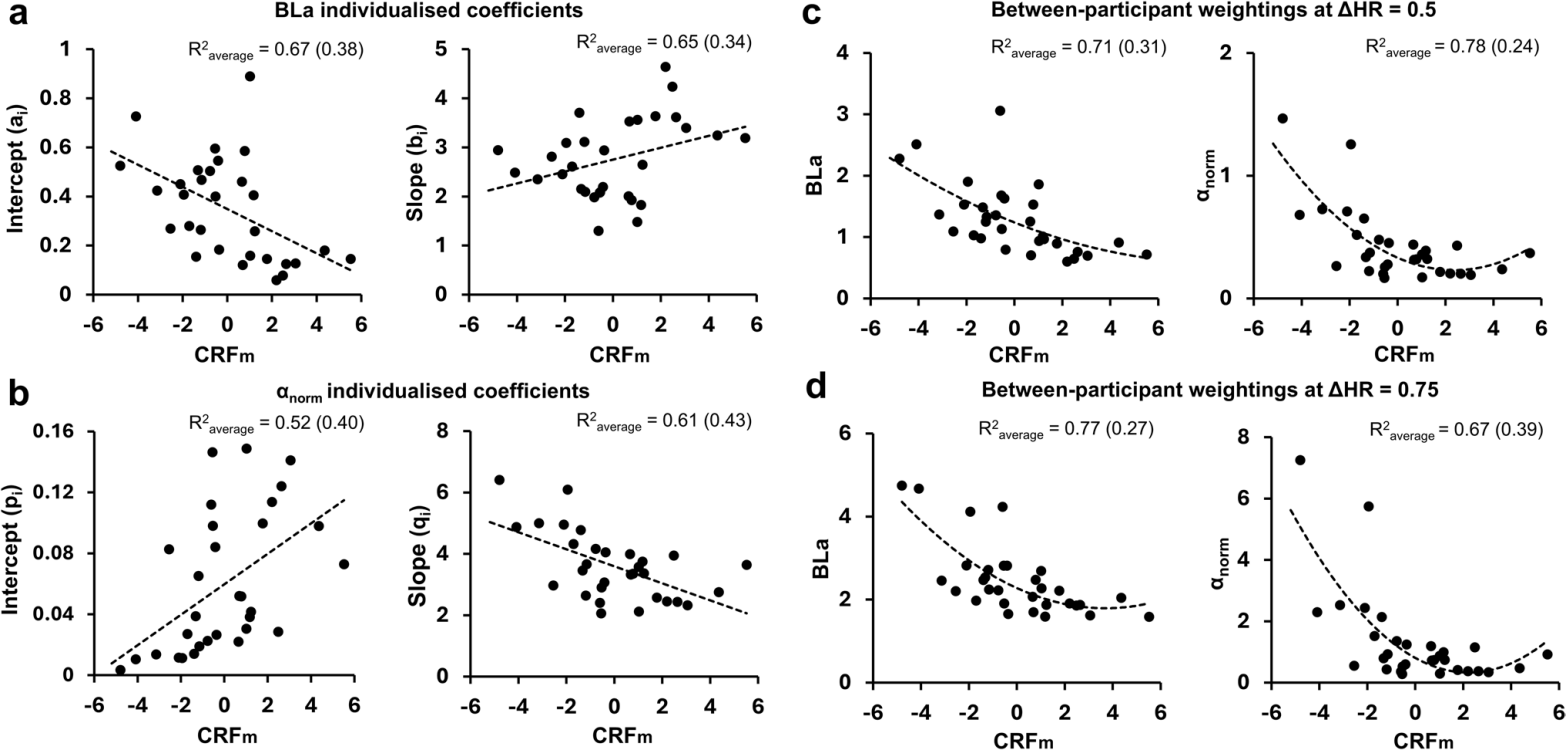
Plots representing the relationship between CRF characteristics (CRF_m_) and **A** iTRIMP individualised coefficients ai and bi, **B** αTRIMP individualised coefficients pi and qi, and (**C** and **D**) iTRIMP/αTRIMP-based weighting factors at ΔHR of 0.5 and 0.75, respectively. *R*^2^_average_ values represented as mean *R*^2^ (± SD). *BLa,* blood lactate; *α*_*norm*_, normalised DFA-α1; *ΔHR,* fractional elevation in HR, *CRF*_*m*_, merged variable for cardiorespiratory fitness characteristics following PCA analysis

The iTRIMP intercept (*a*_*i*_) and slope (*b*_*i*_) coefficients showed a *moderate* relationship with CRF_m_ (*R*^2^_average_ = 0.65–0.67) (Fig. 2A). Similarly, the αTRIMP intercept (*p*_*i*_) and slope (*q*_*i*_) coefficients also showed a *moderate* relationship with CRF_m_ (*R*^2^_average_ = 0.52–0.61) (Fig. 2B). Figure 2C, D displays the relationship between TRIMP weightings (BLa and α_norm_) and CRF_m_ at set internal intensities of ΔHR = 0.5 and 0.75.

## Discussion

The aim was to develop an HRV-based individualised TRIMP (αTRIMP) and examine its construct validity alongside BLa-based iTRIMP by evaluating the relationship between TRIMPs weighting factors and cardiorespiratory fitness (CRF) characteristics. Both iTRIMP and αTRIMP demonstrated to be valid constructs of internal exposure, observed by the inverse relationship between the respective TRIMP weightings and CRF at the same ΔHR. The between-participant variation in CRF was reflected by the between-participant variation in TRIMP weightings at the same heart rate (i.e. a higher weighting factor for lower fitness at the same ΔHR). The αTRIMP shows potential as a practical and accessible method to measure individualised internal training exposure, which can be calculated based on the weighting factors derived from individuals DFA-α1 response to a graded incremental test using a chest-worn HR sensor.

### Construct validity of TRIMP weightings

This is the first study to assess the construct validity of individualised TRIMP methods. Whilst dose–response associations of iTRIMP with adaptation have been reported (Taylor et al. 2018; Ellis et al. 2021), no studies yet have assessed the construct validity of the weighting factors against measures of CRF. This is important to establish, as the weighting factors form the conceptual basis of individualised internal exposure. Both BLa and α_norm_ as weighting factors in the present study exhibited a *moderate* to *strong* relationship with CRF_m_ (*R*^2^_average_ = 0.67–0.78) at fixed ΔHR of 0.5 and 0.75. This is consistent with the rationale behind the development of weighting factors within TRIMPs calculation, which aims to individualise the internal intensity such that individuals with lower fitness would have higher weightings at a standardised ΔHR to account for higher internal exposure. This is indeed observed for both the iTRIMP and αTRIMP methods in the present study.

The iTRIMP coefficients (*a*_*i*_ and *b*_*i*_) and αTRIMP coefficients (*p*_*i*_ and *q*_*i*_) showed a *moderate* relationship (*R*^2^_average_ = 0.52–0.67) with the CRF characteristics. Given the DFA-α1 values are confined in a bounded numerical scale, the differences in DFA-α1 kinetics (slope) with increasing ΔHR dictate the ‘weighted intensity’ between participants instead of the absolute value of DFA-α1 at a given time point like BLa. Hence, the construct of αTRIMP weighting involves the use of *q*_*i*_ as an individualised coefficient, whereas iTRIMP combines both *a*_*i*_ and *b*_*i*_ for generation of its weightings. Future research should explore within-individual changes in individual coefficients of these TRIMP methods to better understand their construct within the weightings and how these coefficients change with training adaptation. It is important to note that following cross-validation, R^2^ for all models of BLa and α_norm_ based weightings and their coefficients with CRF had a relatively large ± SD, indicating that some proportion of variance in weighting factors was still not captured from CRF characteristics. This can partly be attributed to the natural biological variation that exists even within the criterion measure (i.e. CRF) (Pallarés et al. 2016; Sheoran et al. 2024). However, considering the near identical model fit, both iTRIMP and αTRIMP methods show similar confidence to valid constructs of training exposure.

Conventional training exposure methods where internal intensity is expressed solely as fixed percentages of HR or $\dot{V}$O_2max_ fail to consider for fitness-dependent heterogenous metabolic responses between individuals at standardised intensities (Scharhag-Rosenberger et al. 2010; Iannetta et al. 2020). As also observed in Fig. 1A, individual differences exist in relative HR where defined physiological intensity thresholds occur, which warrants the need to weight intensity and avoid misestimation of internal exposure. Practitioners should be wary of using solely HR without weighting to reflect intensity of exercise. Therefore, the weighting factors act as a means of capturing this inter-individual variation in physiological responses at theoretical similar intensities and represent ‘true weighted intensity’ and total internal exposure.

### Generation of αTRIMP

The αTRIMP is calculated from profiling of DFA-α1 response to an incremental test to generate individualised coefficients for weighting factors. Once the coefficients are established, weightings for each ΔHR can be estimated and the total session αTRIMP is calculated. The method generation process is similar to iTRIMP, where individual BLa profiling is carried out to an incremental test and the individualised coefficients generated are used to estimate weighting factors for each ΔHR (Manzi et al. 2009b; Akubat and Abt 2011).

Although lactate is a vital energy substrate that plays a key role in energy metabolism, the BLa response with incremental exercise aims to illustrate the net changes in lactate production and consumption with increasing external intensity (Hall et al. 2016), representative of increasing metabolic demands. Therefore, iTRIMP weighs the exercise intensity (ΔHR) based on an individual’s BLa profile over time when the external intensity is increased. Similarly, the αTRIMP method attempts to weigh the intensity based on DFA-α1 response to an incremental exercise. Based on the concept of “network physiology”, the DFA-α1 response can be understood as a whole-system organismic regulation during exercise represented within the correlation properties of HRV (Rogers and Gronwald 2022). The regulation of parasympathetic and sympathetic branches of ANS, along with other neuromuscular, biochemical, peripheral, and central nervous system inputs, forms the overall concept of “organismic demand” reflected in DFA-α1. Therefore, the DFA-α1-based individualised weightings in αTRIMP aims to capture these changes in organismic demand with increasing ΔHR.

### iTRIMP method considerations

Although iTRIMP was developed over a decade ago, its use is still limited in practice due to its accessibility, time, and cost-effectiveness for its calculation. Besides, the weighting factor within iTRIMP represents an interpolated absolute value of BLa concentration at different intensities based on an incremental test (Eq. 2), which assumes the lactate to be indicative of individualised physiological stress at a given intensity. However, this construct of the iTRIMP weightings should be interpreted with some caution. Firstly, as BLa concentration is a result of complex interaction between muscle lactate production, uptake, and removal by biological tissues, a change in absolute BLa concentration may be a result of these factors and not fundamentally capture the instantaneous physiological stress at that intensity. Secondly, using absolute value of BLa fails to account for inter-individual variability that can exist in the lactate concentration at the same physiological intensity (Kyle et al. 1989; Beneke et al. 2000; Billat et al. 2003). For example, given the workload independent between-individual variation at maximum lactate steady state (MLSS)(Beneke et al. 2000), consider two athletes A and B with relatively similar CRF, exercising at the same relative workload or ΔHR which elicits their respective MLSS, whereby athlete A has an MLSS at 3 mmol L^−1^ and B at 5 mmol L^−1^. Regardless of both athletes A and B exercising at similar relative physiological intensity, the latter would still have a higher weighting factor due to higher absolute value of lactate concentration and therefore greater iTRIMP. Finally, the iTRIMP weightings have also been argued to underestimate the true weighted intensity during intermittent exercise such as in team sports, given the weightings are generated through the ΔHR–BLa relationship during a continuous incremental test (Akubat and Abt 2011).

From a practical application standpoint, αTRIMP is a novel internal exposure measurement generated through HRV-based individualised weightings that can be effectively used within training monitoring. Because the primary component required to calculate αTRIMP weightings is RR interval data derived from a chest-worn ECG sensor, it is an accessible, cost, and time-effective method, particularly in a large team environment where training exposure methods like iTRIMP may not be practically feasible. In addition, as the weightings are likely to vary with changes in fitness levels, it is necessary to have access to a method like αTRIMP where frequent reassessment to update the weightings is viable.

The study findings should, however, be interpreted keeping into account certain limitations. Firstly, the weightings were generated during a laboratory-based incremental test design, which may not apply in the field. Future work is needed to explore the efficacy of field-based incremental tests for generation of DFA-α1-based weightings. In addition, different treadmill protocols (step for iTRIMP and ramp for αTRIMP) were used for TRIMPs generation process, which may affect the BLa/DFA-α1 relationship with HR (Akubat and Abt 2011). Lastly, the present study had a relatively heterogenous sample with respect to CRF characteristics which may influence the model performance with more variability within TRIMP weightings.

Future research is warranted to understand the efficacy of DFA-α1 to weight intensity in elite athletic populations who are expected to have more nuanced and homogenous physiological responses to increasing external intensity. With the present study observing αTRIMP as a valid construct of internal exposure, future work is needed to establish its criterion validity in training dose–response association with training outcomes. In addition, the evidence of construct validity for iTRIMP and αTRIMP weightings should be extended by monitoring the effect of changes in CRF on individualised coefficients and the overall weighting factors.

## Supplementary Information

Below is the link to the electronic supplementary material.

Supplementary file1 (DOCX 103 KB)

## Data Availability

The datasets generated and analysed during the current study are not publicly available as part of Leeds Beckett University Data Management Plan, but will be made available by the corresponding author upon reasonable request.

## Abbreviations

ANS: Autonomic nervous system
*a*_*i*_: Intercept of blood lactate versus ΔHR curve
α_norm_: Normalised detrended fluctuation analysis
αTRIMP: DFA-α1 (normalised)-based training impulse
*b*_*i*_: Slope of blood lactate versus ΔHR curve
BLa: Blood lactate
bTRIMP: Banister’s training impulse
CRF: Cardiorespiratory fitness
*CRF*_*m*_: Combined cardiorespiratory fitness variable from principal component analysis
DFA-α1: Detrended fluctuation analysis
ΔHR: Fractional elevation in HR
ECG: Electrocardiogram
HR: Heart rate
HR_max_: Maximal heart rate
HRV: Heart rate variability
iTRIMP: Individualised training impulse
KMO: Kaiser–Meyer–Olkin
LT_1_: First lactate threshold
LT_2_: Second lactate threshold
MLSS: Maximum lactate steady state
PCA: Principal component analysis
PCR: Principal component regression
PC1: First principal component
*p*_*i*_: Intercept of α_norm_ versus ΔHR curve
*q*_*i*_: Slope of α_norm_ versus ΔHR curve
RPE: Rating of perceived exertion
TRIMP: Training impulse
$\dot{V}$O_2max_: Maximal oxygen uptake
VT_1_: First ventilatory threshold
VT_2_: Second ventilatory threshold
v$\dot{V}$O_2max_: Minimum treadmill speed to elicit maximal oxygen uptake
vVT_1_: Treadmill speed at the first ventilatory threshold
vVT_2_: Treadmill speed at the second ventilatory threshold
vLT_1_: Treadmill speed at the first lactate threshold
vLT_2_: Treadmill speed at the second lactate threshold
*w*_*i*_: Weighting factor based on DFA-α1
*y*_*i*_: Weighting factor based on blood lactate
