# Supplementary material for: Internal training exposure: development and construct validation of an individualised method using heart rate variability
Source: Eur J Appl Physiol. 2025 Jun 11;125(11):3341–50. doi: 10.1007/s00421-025-05841-y (PMC12528351; doi:10.1007/s00421-025-05841-y)
Supplement: Supplementary file 1 — Supplementary file1 (DOCX 103 KB) [file 421_2025_5841_MOESM1_ESM.docx]

**
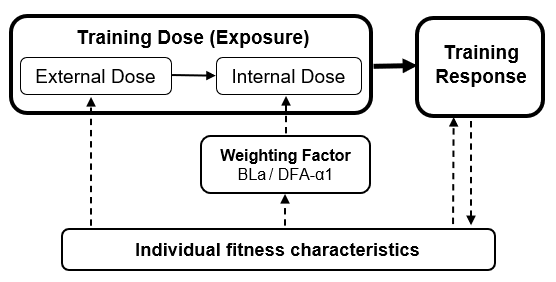
Supplementary Figure 1.** Conceptual training dose-response relationship wherein weighting factors mediate the influence of individual fitness characteristics on the internal dose and thus the overall training exposure. Abbreviations: BLa, blood lactate; DFA-α1; detrended fluctuation analysis of heart rate variability.


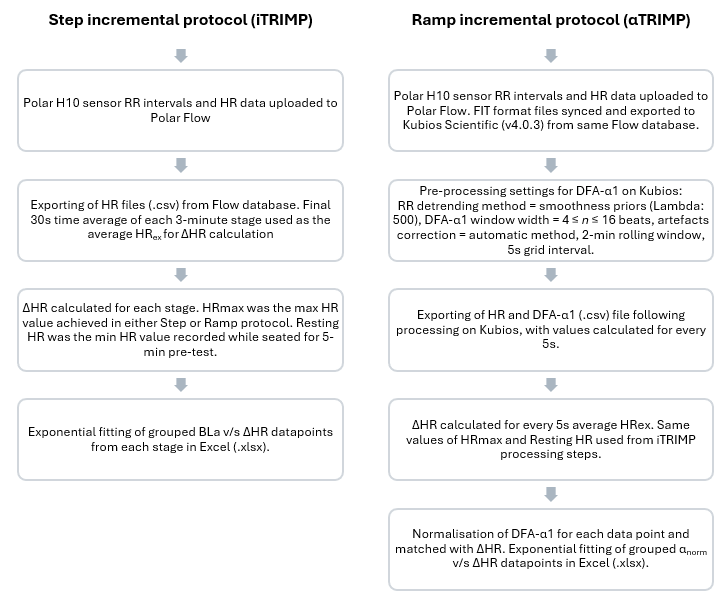


**Supplementary Figure 2.** Schematic representation of the pre- and post-processing steps for HR and RR intervals data during TRIMPs generation process. Abbreviations: BLa, blood lactate; DFA-α1; detrended fluctuation analysis of heart rate variability, ΔHR = fractional elevation in HR.


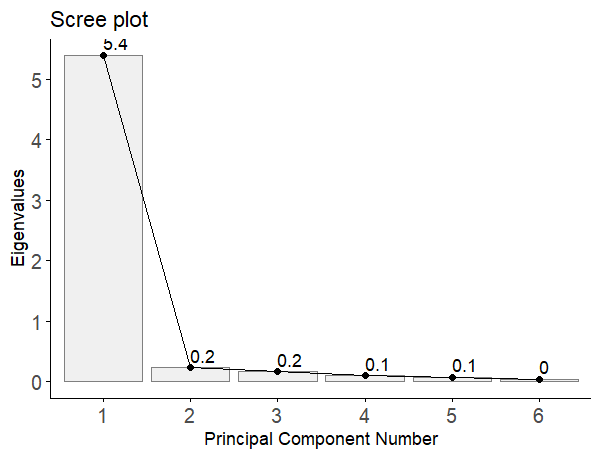
**Supplementary Figure 2.** Scree plot of all 6 principal components (PCs) based on cardiorespiratory fitness variables (vLT_1_, vLT_2_, vVT_1_, vVT_2_, V̇O_2max_ and vV̇O_2max_) with their eigenvalues.

| ***Supplementary Table 1.*** *Specifications of principal component regression models to assess the relationship between cardiorespiratory fitness (PC1) and weighting factors of iTRIMP and αTRIMP (BLa and α_norm_).* | | | | | | | | | | |
| --- | --- | --- | --- | --- | --- | --- | --- | --- | --- | --- |
|  | **Predictors** | | |  | **Model Fit** | | | |  | |
| **Variable** | **Intercept** | **PC1** | **(PC1)^2^** |  | **AIC** | **RMSE** | **R^2^** | **MAE** | **n** |  |
| ***Weightings at standardised ΔHR of 0.5 and 0.75 and cardiorespiratory fitness (PC1) models*** | | | | | | | | | | |
| BLa (ΔHR = 0.5) | 1.24 | -0.16  [-0.23 to -0.08] | 0.01  [-0.01 to 0.03] |  | 46.18 | 0.41 (0.25) | 0.71 (0.31) | 0.34 (0.19) | 31 |  |
| α_norm_ (ΔHR = 0.5) | 0.33 | -0.09  [-0.12 to -0.05] | 0.02  [0.01 to 0.03] |  | -5.18 | 0.18 (0.11) | 0.78 (0.24) | 0.15 (0.07) | 31 |  |
| BLa (ΔHR = 0.75) | 2.28 | -0.26  [-0.36 to -0.16] | 0.04  [0.00 to 0.07] |  | 63.01 | 0.54 (0.31) | 0.77 (0.27) | 0.45 (0.27) | 31 |  |
| α_norm_ (ΔHR = 0.75) | 0.81 | -0.43  [-0.64 to -0.23] | 0.10  [0.04 to 0.15] |  | 77.01 | 1.04 (0.73) | 0.67 (0.39) | 0.78 (0.44) | 31 |  |
| ***Individual coefficients and cardiorespiratory fitness (PC1) models*** | | | | | | | | | | |
| BLa intercept (coefficient a) | 0.39 | -0.05 [-0.10, 0.00] | / |  | 14.17 | 0.23 (0.18) | 0.67 (0.38) | 0.19 (0.11) | 31 |  |
| α_norm_ intercept (coefficient p) | 0.06 | 0.01 [0.00, 0.02] | / |  | -108.09 | 0.04 (0.01) | 0.52 (0.40) | 0.03 (0.01) | 31 |  |
| BLa slope (coefficient *b*) | 2.75 | 0.12  [0.00 to 0.24] | / |  | 75.39 | 0.72 (0.24) | 0.65 (0.34) | 0.64 (0.21) | 31 |  |
| α_norm_ slope (coefficient *q*) | 3.59 | -0.28  [-0.42 to -0.13] | / |  | 86.27 | 0.83 (0.41) | 0.61 (0.43) | 0.72 (0.35) | 31 |  |
| BLa = blood lactate, α_norm_ = DFA-α1 (normalised), PC1 = principal component 1 (or CRF_m_), ΔHR = fractional elevation in HR, AIC = Akaike information criterion, RMSE = root mean square error, MAE = mean absolute error. | | | | | | | | | | |
